# Microparticle dynamics in upper-ocean turbulence: Dataset for analysis, modeling & prediction

**DOI:** 10.1016/j.dib.2024.110850

**Published:** 2024-08-20

**Authors:** Federico Pizzi, Mona Rahmani, Joan Grau, Francesco Capuano, Lluís Jofre

**Affiliations:** a Department of Fluid Mechanics, Universitat Politècnica de Catalunya · BarcelonaTech (UPC), Barcelona 08019, Spain; b Department of Chemical & Biological Engineering, The University of British Columbia, Vancouver, BC V6T 1Z3, Canada

**Keywords:** Aggregation, Direct numerical simulation, Microplastics, Ocean turbulence, Particle-laden flow

## Abstract

Plastic particle pollution has threatened the well-being of seawater ecosystems over the past decades. Therefore, understanding, modeling and (potentially) predicting the dynamics of microplastics and biogenic particles in ocean turbulence is of utmost importance to help develop mitigation strategies and propose technological solutions ultimately aimed at safeguarding global water systems. This is particularly significant for microplastics in the upper-ocean layer. To that end, this work presents a comprehensive and openly accessible dataset carefully designed to explore the interplay between the flow physics of particle-laden turbulence and the physicochemical effects of biofilm stickiness. The dataset comprises nine point-particle direct numerical simulations of fluid flow featuring microplastic and biogenic debris within a periodic three-dimensional flow domain. In all cases, the chosen turbulent intensity and microparticle properties represent conditions observed in the upper-ocean layer. This data repository aims to facilitate in-depth exploration, modeling and prediction of the intricate flow physics observed in marine microplastics, particularly regarding their distribution and aggregation.

Specifications Table

**Table:** 

Subject	Physics
Specific subject area	Fluid mechanics
Type of data	Tables (csv format); Raw, Filtered data; Code & Scripts.
Data collection	A total of 9 simulation runs are acquired through high fidelity direct numerical simulations (DNS). The numerical domain represents an upper-ocean cubic region based on homogeneous isotropic turbulence, along with a bidisperse mixture of point particles representing microplastics and biogenic material. The adopted mesh ensures full resolution of all turbulent scales, and the particle sizes align with literature data. The dataset includes the instantaneous flow velocity field over time, as well as the positions and velocities of the particles. Additionally, the aggregates are computed as a post-process.
Data source location	Dept. Fluid Mechanics*,* Barcelona East School of Engineering, Universitat Politècnica de Catalunya ‧ BarcelonaTech (UPC), Barcelona, Spain
Data accessibility	Repository name: Figshare+ Data identification number: https://doi.org/10.25452/figshare.plus.25498372 Direct URL to data: https://plus.figshare.com/articles/dataset/ Distribution_and_Aggregation_of_Microparticles_in_Upper-Ocean_Turbulence_Dataset/25498372 Instructions: Data are openly accessible and available for download
Related research article	None

## 1 Value of the Data

• This dataset empowers researchers to explore the intricate behavior of microplastic (MP) and biogenic particles (BPs) in the turbulent upper region of oceans through point-particle direct numerical simulation data. It provides time-series data of fully-resolved turbulent flow fields and Lagrangian points which represent the marine debris, facilitating comprehensive analysis and the development of models to predict the fate of microplastics.
• The 9 simulation cases provided, spanning a wide range of particle properties and stickiness characteristics, will be valuable to an interdisciplinary audience including physicists and engineers specialized in fluid mechanics, as well as environmental scientists.
• The innovative integration of physical properties (such as turbulence and particle size) with physicochemical characteristics (e.g., coagulation efficiency) of marine debris enables the calculation of parameters such as particles preferential concentration and distribution, aggregates formation and composition, and the tracking of particle trajectories and collisions.

## 2 Background

The proliferation and impact of marine microplastics, plastic pieces smaller than 5 millimeters, are a growing concern within the realm of environmental science and marine biology [1]. Recent estimations reveal an astonishing presence of plastic debris in the ocean [2], which engage with marine ecosystems [3]. A significant challenge is understanding the fate of “missing plastic”: only 1% of the plastic entering the ocean annually is observed floating at the surface [4]. The prevailing hypothesis attributes this scarcity to the sinking of light plastic debris to deeper layers of the ocean [5] due to the aggregation with biogenic material [5,6]. Nonetheless, examining the aggregation processes of particles in upper-ocean turbulence is particularly challenging due to the complex nature of the problem, involving a multitude of particles, turbulent flow and diverse biophysical properties.

Therefore, motivated by the limited research data available and the growing scientific interest in microplastic behavior in ocean environments, this work is dedicated to present a curated dataset of DNSs comprising 9 particle-laden turbulent cases. By doing so, researchers can pinpoint the precise factors influencing microplastics movement and aggregation, paving the way for more accurate predictive models and helping to develop mitigation strategies.

## 3 Data Description

### 3.1 Dataset hierarchy

The data and post-processing scripts are available as an open dataset [7] on Figshare+ [8]. The 9 different simulations, whose main features are summarized in Table 1, are organized systematically to facilitate access and analysis. The various simulations are characterized by the following properties: for cases 1, 2, 3 and 7, 8, 9, simulations are advanced for five Taylor microscale times (as listed in Table 5), while for cases 4, 5, 6, which contain a significantly large number of particles incurring high computational costs, are advanced for one Taylor microscale time. Such relatively short simulation time is a consequence of the expensive detection of particle-particle collisions algorithm, which as a result imply that particles: (i) do not completely experience the full turbulent spectrum, particularly the large flow scales; and (ii) the effects of gravity become less important as described in Section 4.2. Finally, the parameter that determines whether coalescence occurs upon collision is the stickiness parameter, viz. the probability of particles to attach upon collision events (also defined as coagulation efficiency, *α*) [9,10]. The simulations conducted consider three values of stickiness (*α* = 1/3, 2/3, 3/3) for three different cases of microparticle combinations (see Table 1).

**Table 1:** Summary of input parameters and corresponding dimensionless numbers of the simulations presented in the database. In detail: *d_p1_* represents the diameter of microplastics, *d_p2_* is the diameter of biogenic particles, *n_p1_* denotes the number density of microplastics, *n_p2_* indicates the number density of biogenic particles; *St*_λ1_ is the Stokes number for microplastics, *St*_λ2_ is the Stokes number for biogenic particles; Φ_v_ represents the mixture volume fraction, and *α* is the coagulation efficiency. For a comprehensive description of these parameters, refer to Section 4.

Case	*d_p1_* [m]	*d_p2_* [m]	*n_p1_* [prt/m3]	*n_p2_* [prt/m3]	*St_λ1_*	*St_λ2_*	Φ_v_	*α*
1	1.5‧10^−4^	7.5‧10^−5^	1.0‧10^7^	1.0‧10^7^	1.92‧10^−4^	7.20‧10^−5^	1.99‧10^−5^	1/3
2	1.5‧10^−4^	7.5‧10^−5^	1.0‧10^7^	1.0‧10^7^	1.92‧10^−4^	7.20‧10^−5^	1.99‧10^−5^	2/3
3	1.5‧10^−4^	7.5‧10^−5^	1.0‧10^7^	1.0‧10^7^	1.92‧10^−4^	7.20‧10^−5^	1.99‧10^−5^	3/3
4	1.5‧10^−4^	7.5‧10^−5^	2.5‧10^7^	5.0‧10^7^	1.92‧10^−4^	7.20‧10^−5^	5.52‧10^−5^	1/3
5	1.5‧10^−4^	7.5‧10^−5^	2.5‧10^7^	5.0‧10^7^	1.92‧10^−4^	7.20‧10^−5^	5.52‧10^−5^	2/3
6	1.5‧10^−4^	7.5‧10^−5^	2.5‧10^7^	5.0‧10^7^	1.92‧10^−4^	7.20‧10^−5^	5.52‧10^−5^	3/3
7	3.0‧10^−4^	1.5‧10^−4^	1.0‧10^7^	1.0‧10^7^	7.68‧10^−4^	2.90‧10^−4^	1.59‧10^−5^	1/3
8	3.0‧10^−4^	1.5‧10^−4^	1.0‧10^7^	1.0‧10^7^	7.68‧10^−4^	2.90‧10^−4^	1.59‧10^−5^	2/3
9	3.0‧10^−4^	1.5‧10^−4^	1.0‧10^7^	1.0‧10^7^	7.68‧10^−4^	2.90‧10^−4^	1.59‧10^−5^	3/3

**Fig. 1:**
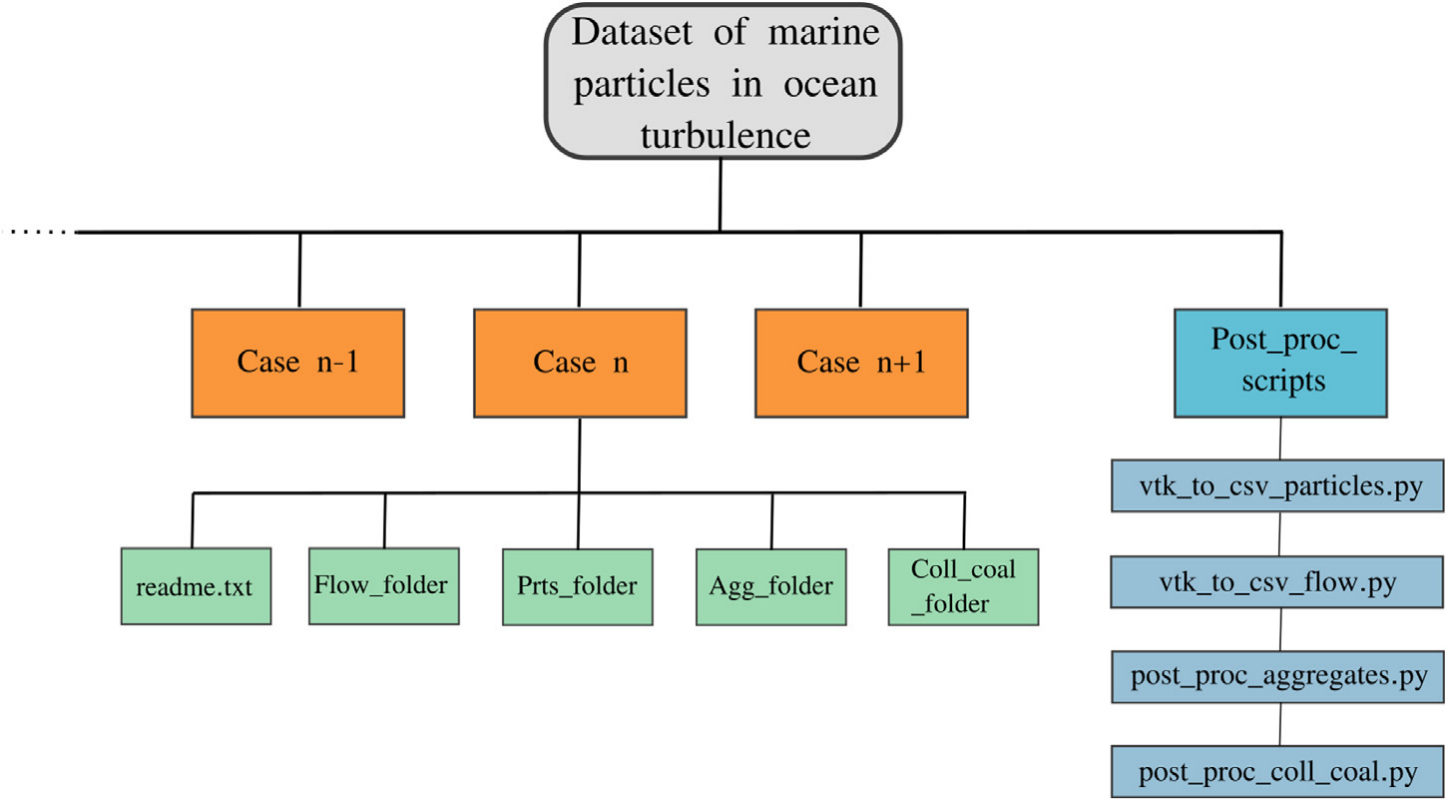
Schematic representing the hierarchical format of the database files. The orange boxes are representative of the 9 cases and for each case the flow, particles, aggregates and collision-coalescence events are stored.

As shown schematically in Fig. 1, each case is stored in a dedicated folder (orange boxes) that, in turn, includes subfolders dedicated to the flow field, particles and aggregates data. Each subfolder contains 50 (cases 1, 2, 3 and 7, 8, 9) or 10 (cases 4, 5, 6) instantaneous snapshots uniformly spaced in time. As specified in Table 2, the flow data (in csv format, named flow_n.csv where n is the ordering number, and located in Flow_folder) includes the x, y, and z velocities (pre-multiplied by the fluid density) and their corresponding coordinates. Table 3 details the properties of the csv particle files (located in Prts_folder and named prt_t_n.csv), which include the x, y, z velocities, the particle class (1 for microplastic or 2 for biogenic particles), and the x, y, z positions. These output variables represent essential parameters directly produced by the simulations, serving as crucial inputs for computing other derived properties.

**Table 2:** Flow phase variables corresponding to the entry columns of the flow data file included in Flow_folder (see Fig. 1). The second column corresponds to the name of the solver output variable printed in the csv files.

Variable	SOLEIL quantity	Symbol	Units
x-velocity	Rho-U_X	*ρ_f_u_x_*	kg/(s‧m^2^)
y-velocity	Rho-U_Y	*ρ_f_u_y_*	kg/(s‧m^2^)
z-velocity	Rho-U_Z	*ρ_f_u_z_*	kg/(s‧m^2^)
x-coordinate	X	*x*	m
y-coordinate	Y	*y*	m
z-coordinate	Z	*z*	m

**Table 3:** Particles phase variables corresponding to the entry columns of the particle data files included in Prts_folder (see Fig. 1).

Variable	SOLEIL quantity	Symbol	Units
x-velocity	Vel : 0	*u_x_*	m/s
y-velocity	Vel : 1	*u_y_*	m/s
z-velocity	Vel : 2	*u_z_*	m/s
Class	dID	-	-
x-coordinate	X	*x_p_*	m
y-coordinate	Y	*y_p_*	m
z-coordinate	Z	*z_p_*	m

On the other hand, as described in Table 4, the post-processed quantities include the properties of the aggregates, such as their composition (i.e., the number of original microplastics and/or biogenic particles), diameter, density, position & velocity.

**Table 4:** List of output (post-processed) aggregate properties/quantities corresponding to the entry columns of the particle data files included in Agg_folder (see Fig. 1).

Description	Field label	Symbol	Units
MPs content	Number prts. 1	-	-
BPs content	Number prts. 2	-	-
Diameter	Aggregate diameter	*d_agg_*	m
Density	Aggregate density	*ρ_agg_*	kg/m^3^
x-coordinate	x	*x_agg_*	m
y-coordinate	y	*y_agg_*	m
z-coordinate	z	*z_agg_*	m
x-velocity	u	*u_agg(x)_*	m/s
y-velocity	v	*u_agg(y)_*	m/s
z-velocity	w	*u_agg(z)_*	m/s

The files containing the information of aggregates, in csv format, are stored in Agg_folder and generated as part of a post-processing routine, which is implemented in the Python file post_proc_aggregates.py (see Fig. 1). In fact, although the particle-flow solver detects and evolves the dynamics of the aggregates through its aggregation kernel, it does not print out the identification or properties of the aggregates. Instead, the solver reports the location and the velocity of all particles, associated to their original identification, even if they are in an aggregate. This is why the particle files presented in Prts_folder contain the same number of rows (viz. particles) for the entire simulation time. Identification of the particles that are in an aggregate at any time can be inferred in the post-processing steps from the overlapping positions of particles reported by the solver. For better clarity, the data for original particles is stored in Prts_folder, and the data for aggregates in the dedicated folder Agg_folder. Moreover, an additional subfolder, named Coll_coal_folder, includes the simulation status file that outlines the temporal progression of collision and coalescence events at each time step. Finally, the simulation setup properties, such as (i) main independent parameters, (ii) dimensionless parameters, and (iii) data saving format, are described in the README.txt file of each case folder.

### 3.2 Usage notes

**Fig. 2:**
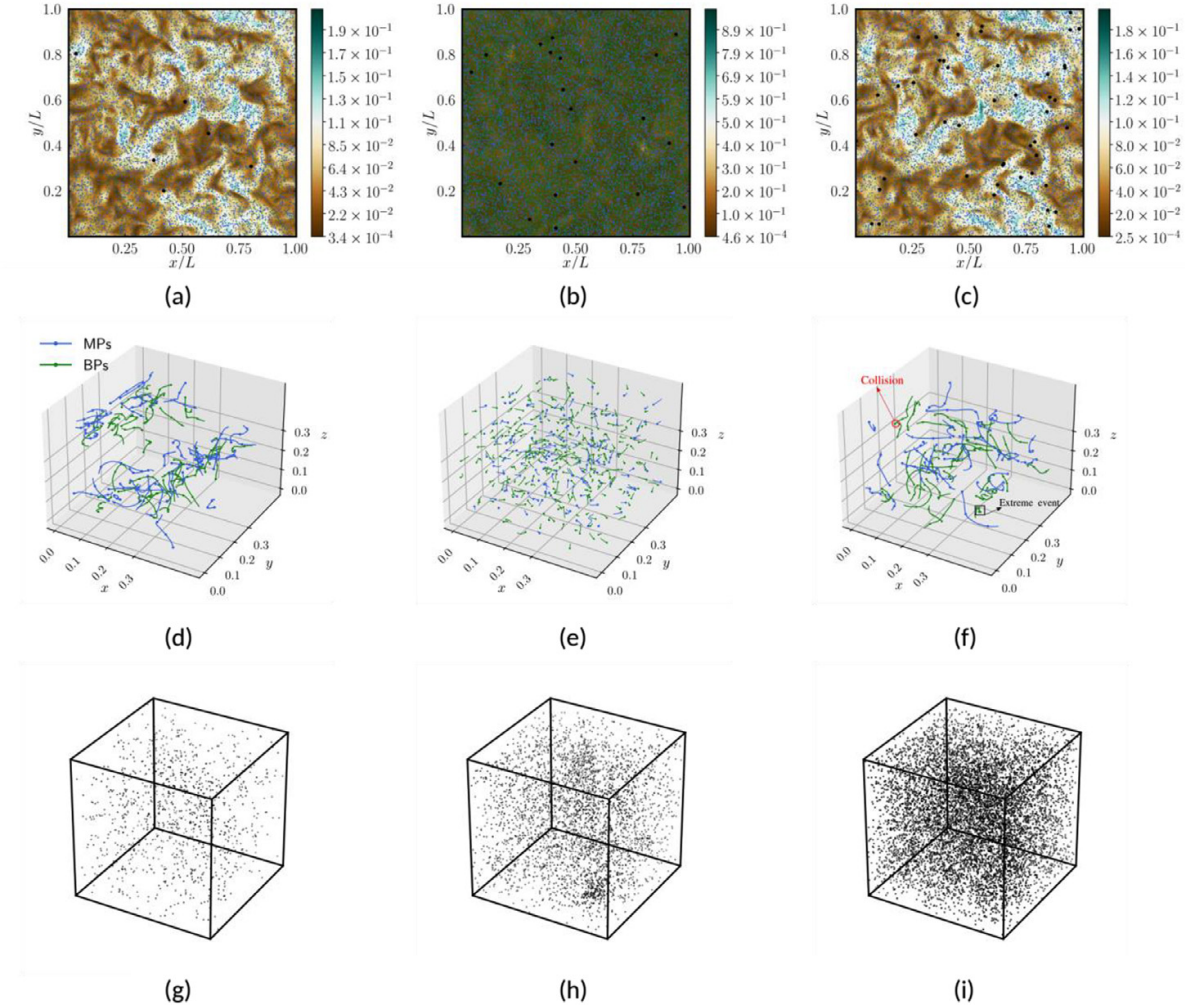
Illustrative post-processed outputs from simulations corresponding to Case 3 (left column), Case 6 (center column) and Case 9 (right column). Plots (a), (b) and (c) depict snapshots of turbulent kinetic energy of the flow, where microplastics, biogenic particles and aggregates are represented as blue, green, and black dots, respectively. Plots (d), (e) and (f) exhibit three-dimensional trajectories of various selected particles/aggregates. Finally plots (g), (h) and (I) showcase snapshots of the aggregates within the 3D domain.

The structured dataset is crafted for user-friendly access, offering readily available data for the instantaneous flow, particles and aggregates files, all stored in csv format. It is important to remark that both microplastic and biogenic particles, as well as their aggregates, are modeled as spherical debris. This simplification enables a greater focus on the overall statistics of marine debris. In detail, each flow file comprises velocity arrays with 196×196×196 data points equally spaced within a periodic domain. The mesh coordinates are labeled as (X, Y, Z) which refer to x, y, and z directions, respectively, as illustrated in Table 4. In terms of particle files, the size of the arrays differs: Cases 1, 2, 3, 7, 8, and 9 consist of 2.5‧10^6^ particles each (corresponding to the number of rows in each file), whereas Cases 4, 5, and 6 contain 9.375‧10^6^ particles each; detailed information about the files are contained in the readme.txt file (see Fig. 1). The comprehensive storage of flow and particle data enables the investigation of complex particle-laden turbulent phenomena. As an example, the user can visualize the particles distribution with respect to the local turbulent intensity as depicted in Figs. 2(a), (b) and (c). For these plots, it is also provided an example of the Python code (see Listing 1) that: (i) reads the flow data and stores them in 3D arrays corresponding to the mesh grid, and (ii) reads the particle data and stores them in z-slices with thickness matching the mesh size. Once this is done, the arrays are ready for plotting. Moreover, the particles output is consistently organized over time, meaning that the particular row of each file corresponds to the same particle. This data setup allows the tracking of particles/aggregates during post-processing analyses, as exemplified in Figs. 2(d), (e) and (f). Such coherence proves highly advantageous as it allows for the examination of geometric properties, visualization of collisions and detection of extreme events, such as small spiral motions of particle trajectories within ocean turbulence. The aggregated files, stored in the Agg_folder in csv format, are generated using an optimized algorithm written in Python language named post_proc_aggregates.py (see Fig. 1). The algorithm used to distinguish aggregates from the original particles is based on basic geometrical considerations and the box-counting method: (i) The box-counting method subdivides the domain into N boxes (6×6×6=216 boxes have been used). For each box, the program checks whether particles are sufficiently close to each other within that subdomain. This approach reduces time consumption since aggregation is a local phenomenon, making it redundant to analyze distances between particles in different boxes. (ii) Once the Euclidean distance between the centers of particle pairs is computed, it is compared to a small threshold value. If the distance is less than this threshold, the aggregate is recorded with a diameter corresponding to (*d_p1_*^3^+ *d_p2_*^3^)^1/3^ and the same operation for density. Aggregates can be visualized such as in Figs. 2(g), (h), (i), or analyzed in a more complex fashion in terms of their dynamics and composition [6].

**List 1:**
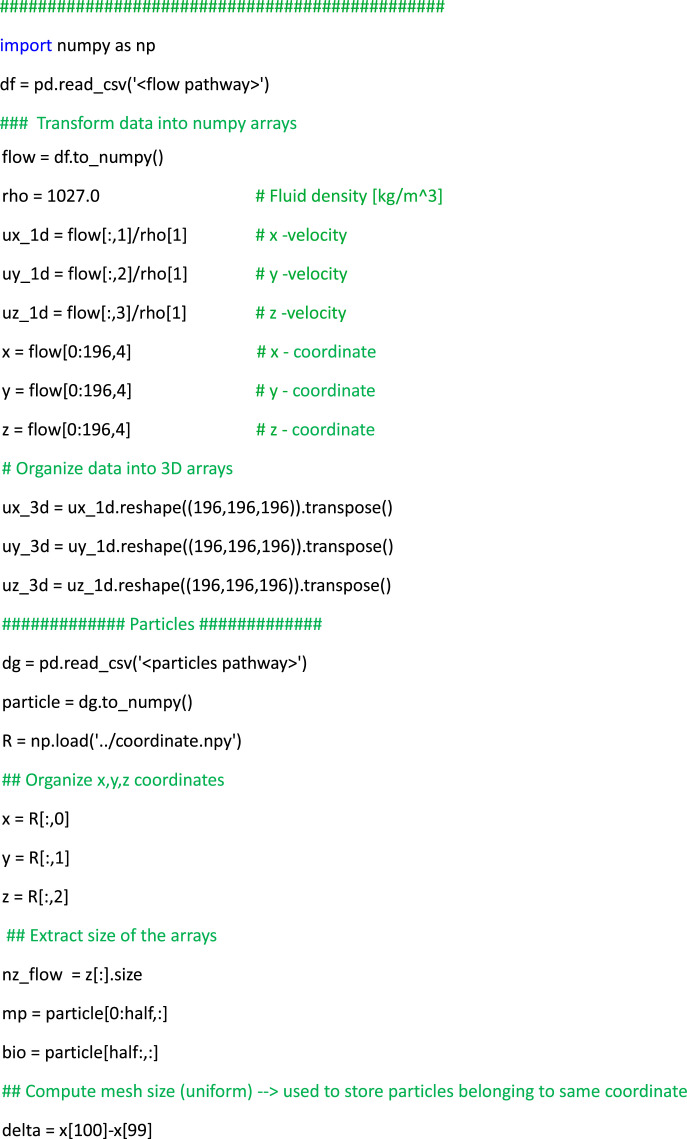

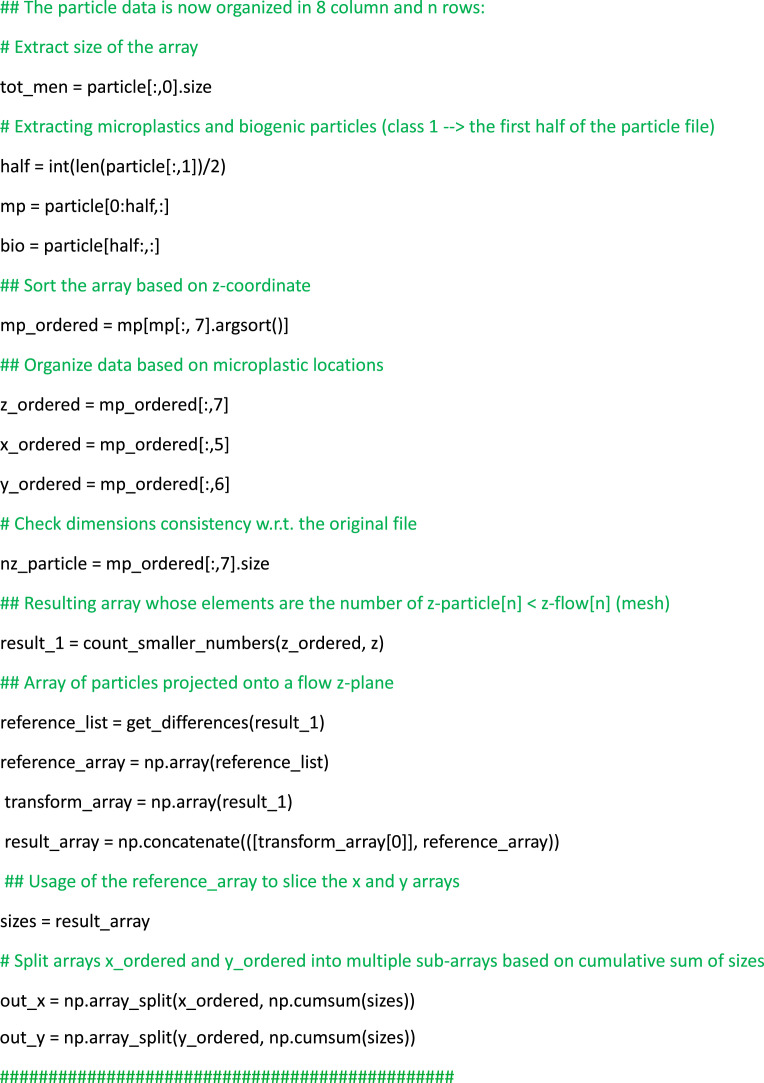
Example of post-processing Python script to read, organize, and plot instantaneous flow and particle data to produce plots similar to Fig. 2(a, b, c).

Finally, an illustrative example of utilizing the dataset to compute the radial distribution function (RDF) of particles, a common metric in particle-laden turbulence used to quantify the preferential concentration of the dispersed phase [13], is showcased. In particular, RDFs can be computed for a set of *M* particles by grouping them based on their separation distance with respect to an isotropic distribution as *g(r) = (N_r_ / V_r_) / (N / V),* where *N_r_* represents the number of particle pairs within a spherical volume element *V_r_* with radius *r* from a given test particle location, *N = M(M-1)/2* denotes the total number of particle pairs within the volume, and V signifies the total volume. Listing 2 provides a simple example of a Python script to calculate the RDF of particles, which is based on computing the volume of spherical shells around each particle and comparing it to an ideal isotropic distribution. This example serves as a practical demonstration of effectively utilizing the particle data for theoretical analyses.

**List 2:**
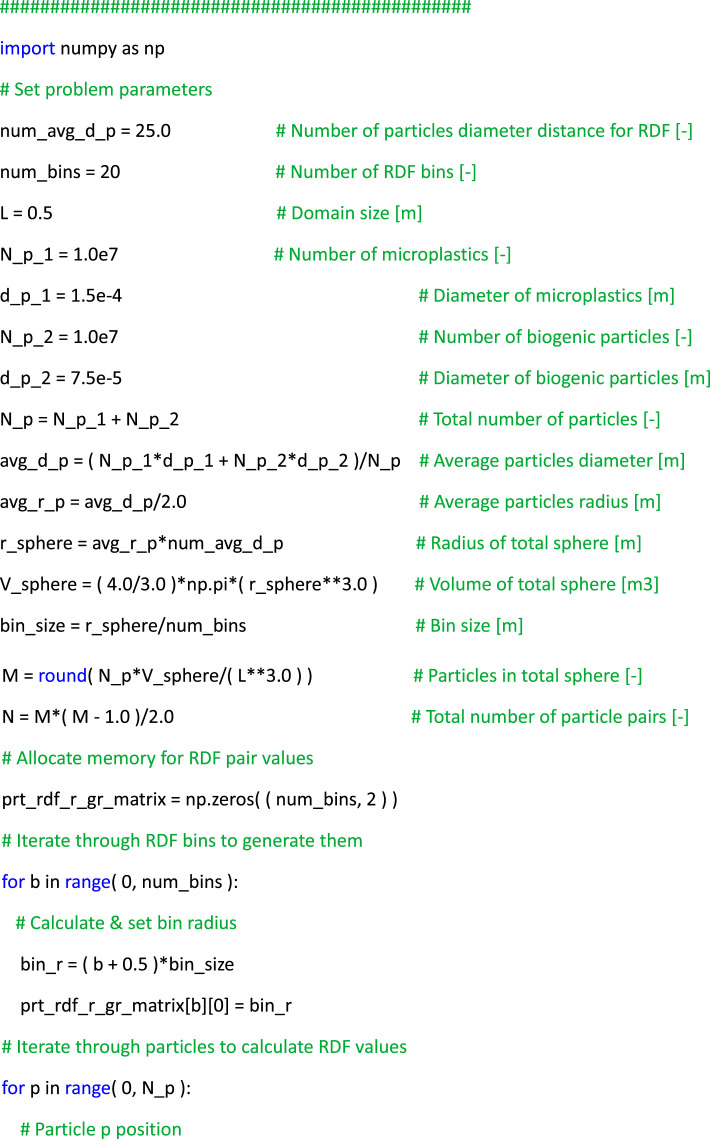

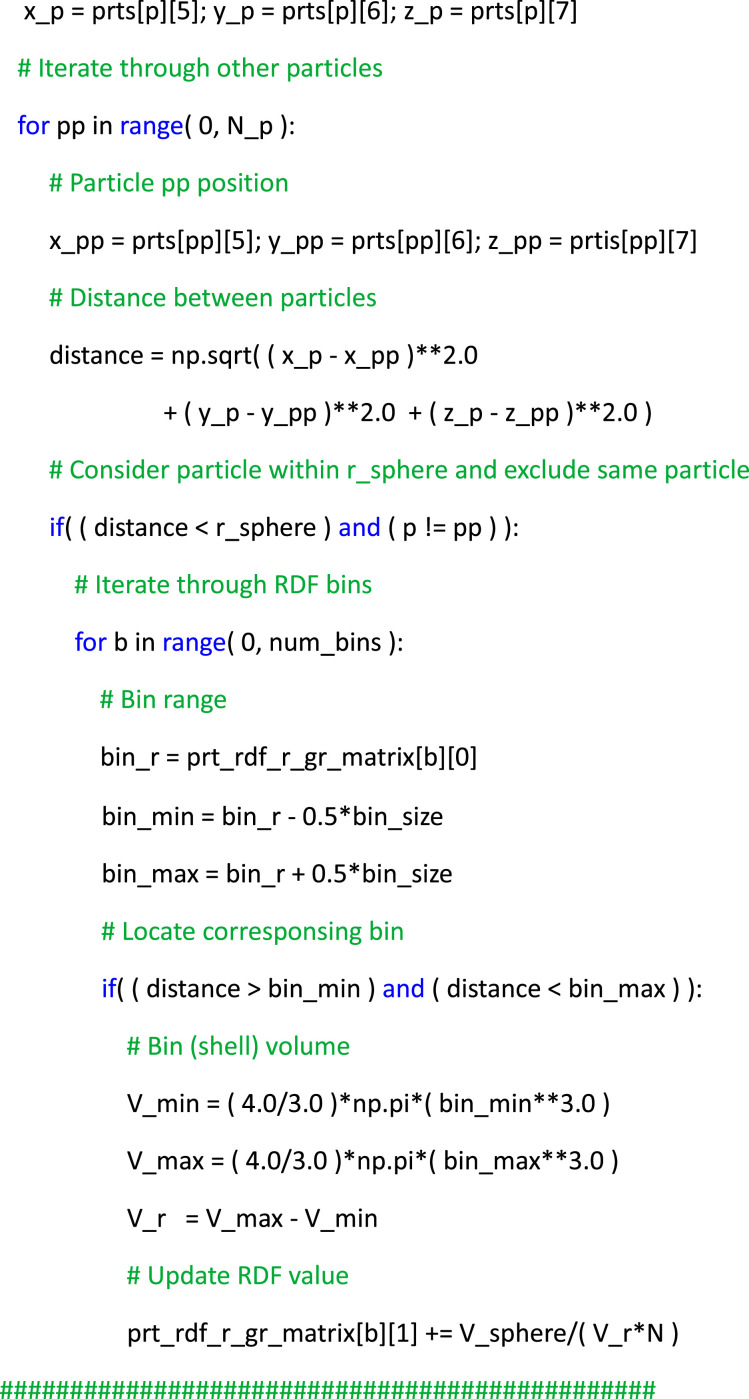
Example of post-processing Python script to read the particle data files and calculate the radial distribution function.

## 4 Experimental Design, Materials and Methods

**Fig. 3:**
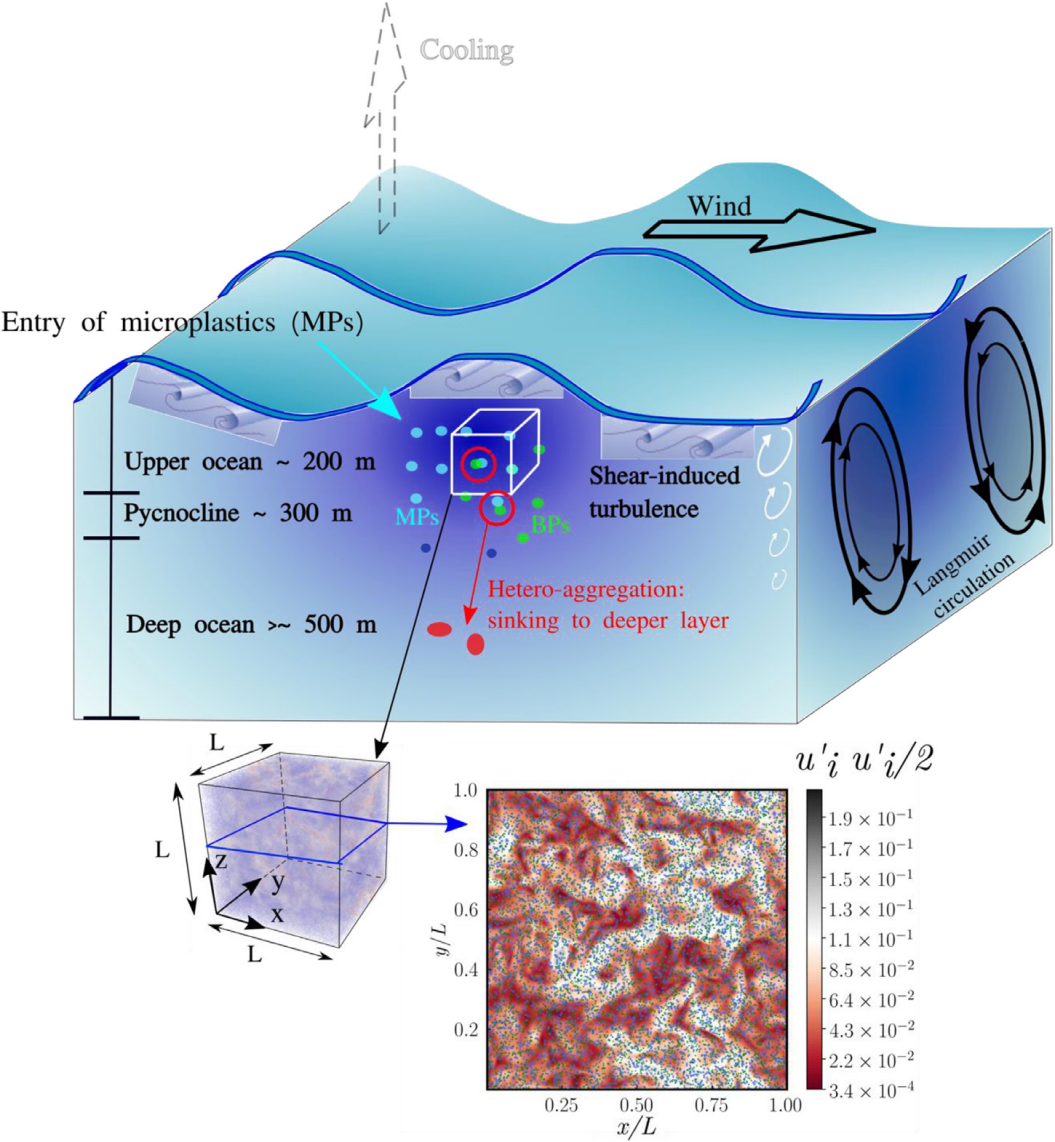
Overview of the primary mechanisms and modeling approach utilized to describe microplastics and biogenic particles in upper-ocean turbulence. The schematic depicts the studied periodic cube containing particles, together with a *x-y* slice visualizing turbulent flow fluctuations and particles distribution. In this representation, biogenic particles are denoted by green points, while microplastics are represented by blue points.

In order to analyze the intricate phenomena of microparticles in oceans, DNS results prove particularly useful as they (i) enable a deep and sophisticated analysis, and (ii) provide access to detailed information of the physics involved. In this regard, the characteristics and strength of oceanic turbulence can vary significantly across different depths, and involve a wide number of processes, as represented in Fig. 3. This work focuses on a small box in the upper-ocean layer which is simulated by means of homogeneous isotropic turbulence (HIT), and neglecting the large-scale shear effects. Under such conditions, marine microplastics can be viewed as an exemplary instance of particle-laden turbulence, wherein turbulent ocean currents (carrier phase) interact with particles (dispersed phase) [9]. Due to the significant separation between flow and particle scales, the representation of microplastics and biogenic material as point particles is motivated by their considerably smaller size compared to the smallest (Kolmogorov) turbulent flow scales.

For this reason, the point-particle (PP) Langrangian-Eulerian (LE) DNS approach is the most natural choice to conduct this type of simulations. In this scenario, particles can be described as *N_p_* small Lagrangian points whose masses are defined as *m_p_* = (π/6) *ρ_p_d^3^_p_*, with *ρ_p_* and *d_p_* denoting their densities and diameters, respectively. Under this premise, the following subsections will focus on the mathematical modeling of the carrier phase, particle description in terms of dynamics and coagulation effects, and finally, the numerical methods utilized for the simulations.

### 4.1 Eulerian description of turbulent flow

Under the assumptions of isothermal, incompressible flow conditions, and by choosing the fluid density *ρ_f_,* dynamic viscosity *μ_f_*, Taylor microscale *λ*, and root-mean-square velocity fluctuations *U_rms_* as scaling parameters [11], the dimensionless continuity and Navier-Stokes equations read [12]

(1) $$\nabla \cdot u_{f}=0,$$

(2) $$\frac{\partial u_{f}}{\partial t}+\nabla \cdot \left(u_{f}⊗u_{f}\right)=-\nabla p+\frac{1}{Re_{\lambda }}\Delta u_{f}+f_{TWC}+F,$$

where $u_{f}$ and p are, respectively, the dimensionless fluid velocity and pressure, and *Re*_λ_ *= λU_rms_* /*ν_f_* is the Taylor Reynolds number with *ν_f_* being the kinematic viscosity of the fluid*.* F represents the normalized forcing term that maintains turbulence at the desired Reynolds number [17]. Further details about this added term are provided in subsection 4.3. The term ***f****_TWC_* corresponds to the two-way coupling term representing the effect of particle forces on the fluid, defined as [9]

(3) $$f_{TWC}=\sum_{k=1}^{N_{s}}\frac{\Phi_{m_{k}}}{St_{\lambda_{k}}N_{p_{k}}}\sum_{p=1}^{N_{p_{k}}}\left(v_{p}-u_{p}\right)\delta \left(x-x_{p}\right),$$

with $u_{p}$ being the dimensionless fluid velocity at particle locations and $v_{p}$ is the particle velocity, *N_s_* the number of particle classes, and $\delta (x-x_{p})$ the Dirac delta function concentrated at the particle positions. The two-way coupling effect is crucial for particle motion (influencing particle dynamics) and for modulating turbulence. However, in dilute suspensions, such as in the current dataset, this effect is virtually negligible [13]. Defining the flow and particles relaxation times as *τ_λ_* = *λ/U_rms_* and *τ_p_* = *ρ_p_d^2^_p_*/(18 *μ_f_*), respectively, the Stokes number *St_λ_* = *τ_p_*/*τ_λ_* based on the Taylor microscale is obtained. Finally, Φ_m_= *ρ_p_*Φ_v_/*ρ_f_* is the mass fraction of particles in the fluid, with Φ_v_ *= N_p_ m_p_ /(ρ_p_ L^3^)* being the corresponding volume fraction.

### 4.2 Lagrangian representation of particles and coagulation efficiency

Consistent with typical conditions encountered in marine environments, particles and aggregates in their early stages of formation are significantly smaller than the Kolmogorov flow scale *η* = (*ν^3^_f_* / *ε*)^1/4^ (where *ε* is the rate of dissipation of turbulent kinetic energy), i.e., *d_p_*/*η* << 1. Furthermore, the influence of settling/rising velocity is neglected as a modeling hypothesis due to the relatively short simulation times compared to the settling time scale *τ_s_* = *λ/U_s_*, where *U_s_* = (1 - *β*) *g* is the settling velocity with *g* the gravitational force and *β =* 3/(2*(ρ_p_* /*ρ_f_)*+1*)* [13] (see Table 5). Moreover, following the values described in the literature [7], the density ratios between particles and fluid are fixed to *ρ_p_* /*ρ_f_* = 0.97 for microplastics and *ρ_p_* /*ρ_f_* = 1.46 for biogenic particles. Particles are consequently modeled following a Lagrangian PP approach in which Stokes' drag $F_{D}$ is the most important force. This assumption is asymptotically valid in the limit in which particles are dense with respect to the fluid. Otherwise, the history force $F_{H}$ of particles needs to be considered. However, $F_{H}$ (i) becomes important only when *ρ_p_* /*ρ_f_* < 10 [13], (ii) has been reported to scale as $\frac{||F_{H}||}{||F_{D}||}∼d_{p}/\eta$ [10], and (iii) typically presents relatively small impacts on the distribution of particles. As a result, Stokes' drag is the force retained and the description of particles in terms of dimensionless positions and velocities is given by

(4) $$\frac{dx_{p}}{dt}=v_{p},$$

(5) $$\frac{dv_{p}}{dt}=\frac{u_{p}-v_{p}}{St_{\lambda_{k}}}$$

**Table 5:** Main timescales of the problem: *τ_λ_* denotes the Taylor microscale time, *τ_η_* represents the Kolmogorov timescale, and *τ_p1_* & *τ_s1_* and *τ_p2_* & *τ_s2_* are the relaxation and settling timescales of microplastics and biogenic particles, respectively. Values in line with Ref. [18].

Timescales	*τ_λ_* [s]	*τ_η_* [s]	*τ_p1_* [s]	*τ_p2_* [s]	*τ_s1_* [s]	*τ_s2_* [s]
Values	4.6	1.2	[4.9: 35] ‧10^−4^	[3.3: 13] ‧10^−5^	[3.1: 13] ‧10^1^	[6.4 : 25]

Particularly, in this work, it is assumed that the particles forming an aggregate are assembled into a spherical, packed configuration, and the resultant aggregate follows the Lagrangian PP equations of motion and collision given by (4)-(5). The density of the aggregate is computed as *ρ_agg_* = Σ_p_ *m_p_* / Σ_p_ *V_p_* and its diameter is calculated as *d_agg_* = (Σ_p_ *d^3^_p_*)^1/3^, where the sums are over all the particles composing the aggregate. Moreover, in the context of PP approximations, interactions between particles are usually characterized using collision models that rely on the conservation of total momentum and energy [6]. The collision dynamics (also referred to as four-way coupling [13]) is described using kinetic models that rely on the equilibrium of overall momentum and energy. Specifically, when two particles (or aggregates) labelled as 1 and 2 engage in a collision, the post-collision (PC) velocities $v_{1PC}$ and $v_{2PC}$ are related to the before-collision (BC) ones as [6]

(6) $$v_{1PC}=\frac{\left[m_{1}v_{1BC}+m_{2}v_{2BC}+m_{2}C_{R}\left(v_{2BC}-v_{1BC}\right)\right]}{\left(m_{1}+m_{2}\right)},$$

(7) $$v_{2PC}=\frac{\left[m_{1}v_{1BC}+m_{2}v_{2BC}+m_{1}C_{R}\left(v_{1BC}-v_{2BC}\right)\right]}{\left(m_{1}+m_{2}\right)},$$

where $v_{1BC}$ and $v_{2BC}$ denote the velocities of the particles before the collision, with *C_R_* being the restitution coefficient. The extremes of *C_R_* correspond to scenarios where the particles merge upon impact (0, indicating a perfectly inelastic collision), or rebound with the same relative velocity as before impact (1, representing a perfectly elastic collision). Intermediate values represent inelastic collisions in which kinetic energy is dissipated.

### 4.3 Computational approach & post-processing

From a computational point of view, the equations of fluid motion (1-2) are discretized using second-order central finite differences implemented in the in-house SOLEIL multiphysics flow solver [14]. A fourth-order Runge-Kutta scheme is used for integrating the equations in time, together with a fractional-step method for imposing conservation of mass [15,16]. The linear system resulting from the Poisson equation utilized to impose mass conservation is solved via a fast Fourier transform (FFT) solver. To ensure fourth-order accuracy, the integration in time of Lagrangian particles (including position, velocity and collision dynamics) is fully coupled with the advancement of the flow equations. Finally, the collision and aggregation of particles are computed by a kernel designed to detect interactions and generate aggregates [6].

The computational domain is of size *L* = 0.5 m containing a mixture of HIT and microparticles and composed of 196×196×196 uniform grid points with resolution η/Δ ∼ O(1) capable to capture the Kolmogorov flow scale. The fluid density and dynamic viscosity are assumed to be *ρ_f_* = 1027 kg/m^3^ and μ_f_ = 1.41‧10^3^ Pa s, respectively, which are representative of seawater at 10° C, as summarized in Table 6. The fluid phase is volumetrically driven by means of a turbulence forcing scheme [17] targeted to produce an average turbulent kinetic energy (TKE) *k* = (3/2) *U^2^_rms_* such that the ratio between domain size and Kolmogorov length scale is *L/η* ∼ O(10^2^), and therefore the small-scale features of the flow are not affected by the triply periodic boundaries. The root-mean-square velocity fluctuations *U_rms_* = 4.3‧10^−3^ m/s and Taylor microscale *λ* = 1.95‧10^−2^ m of the flow are selected to represent wind-induced turbulence with *Re*_λ_ ∼ 60 as characterized by a wind speed of 4 m/s [18]. The dispersed phase is initialized after the flow reaches turbulent steady-state conditions with *N_p1_* and *N_p2_* randomly distributed particles representative of microplastics and biogenic particles, respectively. Following the ranges provided by Eerkes et al. [19] the density of microplastics is set to *ρ_p1_* = 1000 kg/m^3^ and the density of biogenic particles is prescribed to *ρ_p2_* = 1500 kg/m^3^, while the diameters and number densities of particles are chosen to vary for the different cases as described in Table 1. As a result, the Stokes numbers of the particles for the different cases remain inherently small. Table 1 also provides the values of mixture volume fraction, which are calculated as Φ_v_ = (*N_p1_* Φ_v1_ + *N_p2_* Φ_v2_) / (*N_p1_* + *N_p2_*). Integration in time of flow and particles follows a Courant-Friedrichs-Lewy (CFL) condition of CFL = 0.3. Under these conditions, the computational time step corresponds roughly to 2.5‧10^−5^ s, resulting in 184800 iterations per Taylor timescale (refer to the Table 5). The computational resources needed to simulate these cases are substantial, typically ranging from 384 (for cases with relatively low particle number density) to as high as 712 processors for those with larger particle number density and spanning a computation period of approximately 20 days. Consequently, generating the database has necessitated roughly 1.5 million hours of compute time. Other post-processing scripts, meticulously optimized to extract primary variables and compute derived quantities, are provided in the Post_proc_Scripts folder (refer to Fig. 2).

**Table 6:** Physical properties of upper-ocean water and corresponding turbulent flow scales. Values in line with Ref. [18].

Fluid & Flow properties	*μ_f_* [Pa‧s]	*ρ_f_* [kg/m^3^]	*U_rms_* [m/s]	*L* [m]	*λ* [m]	*η* [m]
Marine water	1.41‧10^3^	1027	4.23‧10^−3^	4.9‧10^−1^	1.95‧10^−2^	1.28‧10^−3^

### 4.4 Technical validation

The multiphysics computational flow solver SOLEIL [14], the scientific tool used to create the present database, has undergone an extensive series of development, verification, and validation campaigns. Among them, the solver has been employed to investigate the interaction between turbulent flow and polydisperse particles within the Predictive Science Academic Alliance Program (PSAAP) II at Stanford University, as detailed in the dedicated web-page [20] where noteworthy publications including (i) advancements in the fundamental understanding and characterization of flow mechanisms, (ii) comparative analyses between experimental and computational studies, (iii) subgrid-scale (SGS) modeling of carrier and dispersed phases in large-eddy simulation (LES) approaches, (iv) efficient propagation and quantification of uncertainties, (v) techniques for compression and reduction of high-dimensional flow systems and (vi) validation efforts against canonical particle-laden turbulent flow problems are reported. Recently, the solver has also been expanded to explore the motion and aggregation of microplastics in oceanic turbulence through the implementation of specialized kernels meticulously designed to identify particle interactions and aggregates formation [6].

**Fig. 4:**
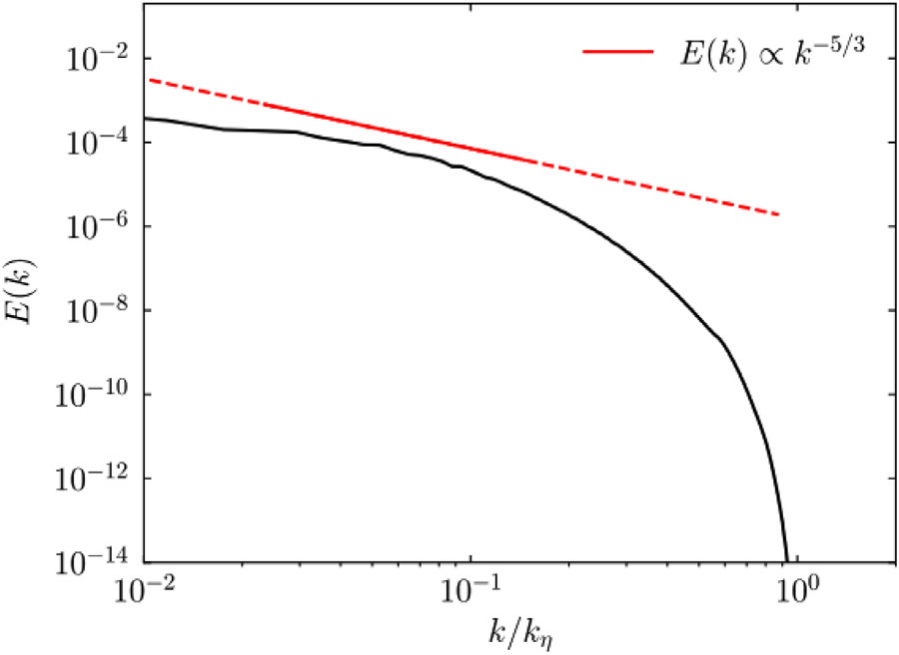
Normalized turbulent kinetic energy spectrum *E(k)* as a function of wavenumber *k* normalized by Kolmogorov wavenumber *k*_η_. The red line indicates a *−5/3* scaling.

For the present computational experiments, crucial validations have been performed to (i) attain adequate mesh resolution in all three spatial dimensions, and (ii) properly characterize the turbulent flow behavior found in upper-ocean layers. Particularly, to validate the computational accuracy/resolution of the flow model utilized, Fig. 4 depicts the normalized turbulent kinetic energy spectrum *E(k)* as a function of normalized wavenumber *k*. As it can be observed from the plot, the energy spectrum reveals two key points: (i) all the important flow scales are sufficiently resolved, as evidenced by the spectrum reaching the Kolmogorov wavenumber *k*_η_; and (ii) the classical -5/3 scaling behavior of HIT in the inertial subrange is recovered.

### 4.5 Code availability

The SOLEIL multiphysics flow solver is accessible as open-source [14].

## Limitations

The limitation of the current dataset lies in the short simulation times typical of turbulence studies. This may hinder the analysis of the fate of large aggregates over the longer time scales associated with ocean currents, which eventually lead to the formation of marine snow. Another constraint of the short simulation time computed is that particles essentially lack the time to experience the impact of large flow scales and gravity effects, which are therefore excluded from the Lagrangian point particle model and should be considered in future studies. Additionally, the particle model does not consider shape characteristics, such as the impact of fiber or disk microplastics. Nonetheless, as a preliminary open-access data source, the “Distribution and Aggregation of Microparticles in Upper-Ocean Turbulence Dataset” aims to encourage researchers to explore the complex physical behavior of microparticles in the ocean.

## Ethics Statement

This work meets ethics requirements and does not involve human subjects, animal experiments, or any data collected from social media platforms and the material is the authors own original work, which has not been previously published elsewhere.

## CRediT authorship contribution statement

**Federico Pizzi:** Conceptualization, Data curation, Formal analysis, Investigation, Writing – original draft. **Mona Rahmani:** Conceptualization, Investigation, Writing – review & editing. **Joan Grau:** Conceptualization, Investigation, Writing – review & editing. **Francesco Capuano:** Conceptualization, Investigation, Funding acquisition, Writing – review & editing. **Lluís Jofre:** Conceptualization, Investigation, Funding acquisition, Writing – review & editing.

## Data Availability

Distribution and Aggregation of Microparticles in Upper-Ocean Turbulence Dataset (Original data) (Figshare+).
